# Elimination and eradication goals for communicable diseases: a systematic review

**DOI:** 10.2471/BLT.23.289676

**Published:** 2023-09-06

**Authors:** Laila Khawar, Basil Donovan, Rosanna W Peeling, Rebecca J Guy, Skye McGregor

**Affiliations:** a The Kirby Institute, University of New South Wales Sydney, Wallace Wurth Building (C27), Kensington, New South Wales 2052, Australia.; b London School of Hygiene and Tropical Medicine, London, England.

## Abstract

**Objective:**

To consolidate recent information on elimination and eradication goals for infectious diseases and clarify the definitions and associated terminology for different goals.

**Methods:**

We conducted a systematic search of the World Health Organization’s Institutional Repository for Information Sharing (WHO IRIS) and a customized systematic Google advanced search for documents published between 2008 and 2022 on elimination or eradication strategies for infectious conditions authored by WHO or other leading health organizations. We extracted information on names of infectious conditions, the elimination and eradication goals and timelines, definitions of goals, non-standardized terminology, targets and assessment processes.

**Findings:**

We identified nine goals for 27 infectious conditions, ranging from disease control to eradication. In comparison with the hierarchy of disease control, as defined at the Dahlem Workshop in 1997, six goals related to disease control with varying levels of advancement, two related to elimination and one to eradication. Goals progressed along a disease-control continuum, such as end of disease epidemic to pre-elimination to elimination as a public health problem or threat. We identified the use of non-standardized terminology with certain goals, including *virtual* elimination, *elimination* of disease epidemics, public health *threat* and public health *concern.*

**Conclusion:**

As we approach the 2030 target date to achieve many of the goals related to disease control and for other infections to become candidates for elimination in the future, clarity of definitions and objectives is important for public health professionals and policy-makers to avoid misperceptions and miscommunication.

## Résumé

**Objectif:**

Rassembler les informations récentes concernant les objectifs d'élimination et d'éradication des maladies infectieuses, mais aussi clarifier la terminologie et les définitions associées aux différents objectifs.

**Méthodes:**

Nous avons mené une recherche systématique au sein des archives institutionnelles pour l'échange d'informations (IRIS) de l'Organisation mondiale de la Santé (OMS), ainsi qu'une recherche systématique personnalisée dans Google Advanced Search, afin de trouver des documents publiés entre 2008 et 2022 portant sur les stratégies d'élimination ou d'éradication de maladies infectieuses et rédigés par l'OMS ou par d'autres organisations sanitaires majeures. Nous avons ensuite extrait les informations relatives aux noms des maladies infectieuses, aux objectifs et délais d'élimination ou d'éradication, à la définition des objectifs, à la terminologie non standardisée, aux orientations et aux processus d'évaluation.

**Résultats:**

Nous avons identifié neuf objectifs pour 27 maladies infectieuses, allant de la lutte contre la maladie à son éradication. Selon la classification du contrôle des maladies telle que définie lors de l'Atelier Dahlem en 1997, six objectifs étaient liés à la lutte contre les infections à divers degrés d'avancement, deux à l'élimination et un à l'éradication. Ces objectifs évoluaient dans un continuum de lutte contre les maladies, par exemple de la fin d'une épidémie à l'élimination de la maladie en tant que problème ou menace pour la santé publique, en passant par sa pré-élimination. Nous avons constaté l'emploi d'une terminologie non standardisée pour certains objectifs: élimination *virtuelle*, *élimination* d'épidémies, *menace* sanitaire et *problème *de santé publique notamment*.*

**Conclusion:**

À mesure que nous nous rapprochons de l'échéance de 2030 en matière de réalisation des objectifs de lutte contre les maladies, et pour que d'autres infections puissent prétendre à une élimination dans le futur, il est crucial de clarifier les définitions et les perspectives afin d'éviter toute erreur de communication et de perception chez les professionnels de la santé publique et les responsables politiques.

## Resumen

**Objetivo:**

Consolidar la información reciente relativa a los objetivos de eliminación y erradicación de enfermedades infecciosas, y aclarar las definiciones y la terminología asociada a los diferentes objetivos.

**Métodos:**

Llevamos a cabo una investigación sistemática del Repositorio Institucional para el Intercambio de Información de la Organización Mundial de la Salud (IRIS OMS) y una Búsqueda sistemática Avanzada en Google de documentos publicados entre 2008 y 2022 que versaban sobre las estrategias de eliminación y erradicación de enfermedades infecciosas. Estos documentos habían sido redactados por la OMS o por importantes organizaciones sanitarias. Recabamos información acerca de nombres de enfermedades infecciosas, objetivos y plazos de eliminación y erradicación, definiciones de objetivos, terminología no estandarizada, así como fines y procesos de evaluación.

**Resultados:**

Identificamos nueve objetivos para 27 enfermedades infecciosas, desde el control de dichas enfermedades hasta su erradicación. En función de la jerarquía de control de enfermedades, según se definió en el Taller de Dahlem en 1997, existían seis objetivos relacionados con el control de enfermedades, que contaban con diferentes niveles de avance; dos objetivos relacionados con la eliminación, y uno que trataba sobre la erradicación. Los objetivos evolucionaron hacia un continuum en el control de enfermedades, desde el fin de la enfermedad epidémica a la preeliminación y la eliminación como problema o amenaza pública sanitaria. Detectamos el uso de terminología no estandarizada en determinados objetivos. Ejemplos de esta terminología son eliminación *virtual*, *eliminación* de enfermedades epidémicas, *amenaza* pública sanitaria y *preocupación* pública sanitaria.

**Conclusión:**

Dado que nos acercamos a la fecha límite establecida de 2030 para lograr numerosos objetivos relacionados con el control de enfermedades y con otras afecciones que puedan ser objeto de eliminación en el futuro, es crucial mantener la claridad en las definiciones y en los objetivos para que los profesionales públicos sanitarios y las personas encargadas de la elaboración de políticas no hagan uso de ideas erróneas y no haya problemas de comunicación.

## ملخص

**الغرض:**

تعزيز المعلومات الحديثة عن أهداف القضاء على الأمراض المعدية واستئصالها، وتوضيح التعريفات والمصطلحات المرتبطة بها للأهداف المختلفة.

**الطريقة:**

قمنا بإجراء بحث منهجي في المستودع المؤسسي لتبادل المعلومات التابع لمنظمة الصحة العالمية (WHO IRIS)، وبحث منهجي متقدم مخصص على Google عن المستندات المنشورة بين عامي 2008 و2022، بشأن استراتيجيات القضاء على الأمراض المعدية أو استئصالها، والتي تم تأليفها بواسطة منظمة الصحة العالمية، أو غيرها من المنظمات الصحية الرائدة. وقمنا باستخراج معلومات عن أسماء الحالات المعدية، والأهداف والجداول الزمنية للقضاء والاستئصال، وتعريفات الأهداف، والمصطلحات غير القياسية، والأهداف، وعمليات التقييم.

**النتائج:**

لقد حددنا تسعة أهداف لـ 27 من الحالات المعدية، تتراوح من مكافحة الأمراض إلى استئصالها. بالمقارنة مع التسلسل الهرمي لمكافحة الأمراض، كما تم تعريفه في ورشة عمل Dahlem في عام 1997، فإن هناك ستة أهداف تتعلق بمكافحة الأمراض بمستويات متفاوتة من التقدم، وهدفين يتعلقان بالقضاء على الأمراض، وهدف واحد يتعلق بالاستئصال. تقدمت الأهداف على مدى سلسلة متواصلة من مكافحة الأمراض، مثل نهاية الوباء المرضي، إلى ما قبل القضاء عليه، إلى القضاء عليه كمشكلة أو تهديد للصحة العامة. لقد حددنا استخدام المصطلحات غير القياسية مع أهداف معينة، بما في ذلك القضاء الظاهري، والقضاء على الأوبئة المرضية، والتهديد على الصحة العامة، ومخاوف الصحة العامة.

**الاستنتاج:**

مع اقترابنا من الموعد المستهدف لعام 2030 لتحقيق العديد من الأهداف المتعلقة بمكافحة الأمراض، ولكي تصبح أنواع العدوى الأخرى مرشحة للقضاء عليها في المستقبل، فإن وضوح التعريفات والأهداف يعد أمرًا مهمًا للمتخصصين في مجال الصحة العامة وواضعي السياسات لتجنب المفاهيم الخاطئة ومشاكل الاتصالات.

## 摘要

**目的:**

旨在整理有关消除和根除传染病目标的最新信息，并阐明不同目标的定义和相关术语。

**方法:**

我们对世界卫生组织信息共享机构库 (WHO IRIS) 进行了系统搜索，并利用谷歌高级搜索功能对世界卫生组织或其他主要卫生组织在 2008 年至 2022 年间发表的传染病消除或根除策略相关文件进行了自定义系统搜索。我们提取了与传染病名称、消除和根除目标及时间表、目标定义、非标准化术语、目标以及评估过程有关的信息。

**结果:**

从疾病控制至根除，我们确定了 27 种传染病的九个目标。与 1997 年达勒姆研讨会上确定的疾病控制等级相比，这些目标包括进展程度各不相同的六个疾病控制相关目标，两个传染病消除相关目标以及一个传染病根除相关目标。按疾病控制流程逐步实现目标，例如首先防止被视为公共卫生问题或威胁的传染病继续蔓延，然后采取预先消除措施，最后实现疾病根除。我们确定了在实现某些目标的过程中需使用的非标准化术语，包括*虚拟*消除、流行病*消除*、公共卫生*威胁*和公共卫生*问题*。

**结论:**

随着涉及实现许多疾病控制相关目标的 2030 年目标日期逐渐临近，为了未来将更多其他传染病纳入消除范围，必须明确定义和目标，以免公共卫生专业人员和决策者产生误解和理解错误。

## Резюме

**Цель:**

Обобщить последнюю информацию о целях по ликвидации и искоренению инфекционных болезней, уточнить определения и сопутствующую терминологию для различных целей.

**Методы:**

Проведен систематический поиск в Институциональном хранилище Всемирной организации здравоохранения для обмена информацией (WHO IRIS) и специализированный систематический расширенный поиск в Google по документам, опубликованным в период с 2008 по 2022 год и посвященным стратегиям по ликвидации или искоренению инфекционных заболеваний, авторами которых являются специалисты ВОЗ или других ведущих организаций здравоохранения. Кроме того, была собрана информация о названиях инфекционных заболеваний, целях и сроках ликвидации и искоренения, определениях целей, нестандартизированной терминологии, целевых показателях и процессах оценки.

**Результаты:**

В отношении 27 инфекционных заболеваний определены девять целей: от борьбы с заболеванием до его искоренения. По сравнению с иерархией контроля заболеваний, определенной на Далемском семинаре в 1997 г., к контролю заболеваний с разной степенью продвижения относятся шесть целей, две из которых связаны с ликвидацией и одна – с искоренением. В ходе реализации этих целей осуществляется непрерывный контроль над заболеванием, например от окончания эпидемии до предварительной ликвидации и устранения проблемы или угрозы общественному здравоохранению. Авторы отмечают использование нестандартизированной терминологии с определенными целями, среди которых *фактическая* ликвидация, *ликвидация* эпидемий заболеваний, *угроза* общественному здоровью и *проблемная ситуация* в области общественного здоровья.

**Вывод:**

По мере приближения к 2030 году, когда должны быть достигнуты многие цели, связанные с контролем заболеваний, а другие инфекции в перспективе станут кандидатами на ликвидацию, специалистам в области общественного здравоохранения и директивным органам важно четко сформулировать определения и цели во избежание неправильного восприятия и недопонимания.

## Introduction

In the past two decades, strong political and financial commitments have led to remarkable national and regional achievements in controlling communicable diseases. The World Health Organization (WHO) has called the final decade of the sustainable development goals (SDGs) a decade for disease elimination.^1^ To meet the 2030 targets of ending long-term epidemics of infectious diseases, such as human immunodeficiency virus (HIV), tuberculosis, viral hepatitis and neglected tropical diseases, requires an integrated response. It is important that these initiatives are developed and clearly communicated using standardized terminology.

WHO leads the intergovernmental community in tackling global health challenges, and takes direction on setting priorities from its 194 Member States, and other technical and financial partners. The World Health Assembly, the decision-making body of WHO, which is attended by delegations from the Member States, passes resolutions with specific health objectives, including those on disease control, elimination and eradication.^2^^,^^3^ The first and only human communicable disease targeted for eradication for which this goal has been achieved is smallpox, in 1977.^4^ Some eradication programmes have been unsuccessful, such as for malaria, hookworm and yellow fever.^5^ Nonetheless, these setbacks have contributed to our understanding of the complexities of disease eradication. Subsequently, the World Health Assembly called for other goals, such as the elimination of certain infectious diseases as a public health problem. With the increasing number of disease-control goals, one of the aims of the Dahlem Workshop in 1997 was to outline the hierarchy of definitions of disease control (Box 1)^3^ These definitions have been embedded in the strategic recommendations of WHO.

Box 1 **Definitions of disease control, elimination and eradication**

***Disease control***

Reduction of disease incidence, prevalence, morbidity and/or mortality to a locally acceptable level as a result of deliberate efforts. Continued actions are required to maintain the decrease. Example: diarrhoeal diseases

***Elimination of disease***

Reduction to zero of the incidence of a specified disease in a defined geographical areas as a result of deliberate efforts. Continued actions are required to prevent re-establishment. Example: neonatal tetanus

***Elimination of infection***

Reduction to zero of the incidence of infection caused by a specific agent in a defined geographical areas as a result of deliberate efforts. Continued actions are required to prevent re-establishment. Example: measles

***Eradication***

Permanent reduction to zero of the worldwide incidence of infection caused by a specific pathogen as a result of deliberate efforts; intervention measures are no longer needed. Example: smallpox

Source: Dowdle WR, 1998.^3^

Elimination of any infectious disease is an ambitious strategy requiring substantial resources to succeed. Often, as the occurrence of an infection falls, further resources are needed to reach the most marginalized or vulnerable subgroups. A perception that a disease has been eliminated, when in fact it has just declined and transmission is still occurring locally, could have serious unintended consequences. For example, commitment and funding from donors may fall or preventive attitudes and behaviours in the community may change, leading to re-emergence of the disease. This danger is illustrated by the resurgence of tuberculosis in affluent countries in the 1990s due to overly confident predictions that led to decreased public health expenditure.^6^^,^^7^ More recently, the goal of ending the acquired immunodeficiency syndrome (AIDS) epidemic has been formulated differently in different country strategies. For example, Australia aims to “virtually eliminate” HIV or “end HIV transmission” by 2025,^8^^,^^9^ and England aspires to end or eliminate or eradicate HIV transmission by 2030.^10^^,^^11^ It is unclear if the end goal of these strategies is ending the AIDS epidemic or if they have more optimistic targets, and if the terms such as virtual elimination, interruption of transmission and elimination of transmission can be used interchangeably. Consistency in terminology and definitions is crucial to reduce misperceptions and ensure uniformity of appropriate goals and outcomes.

Previous reviews described the inconsistencies in the language around disease control initiatives.^12^ However, they did not systematically examine the terminology used for all infections and infectious diseases (hereafter called infectious conditions) targeted for elimination or eradication. In this systematic review we aim to: (i) describe the elimination and eradication goals set by WHO in relation to their definitions in Box 1; (ii) identify inconsistent terminology to facilitate the use of a standardized approach in developing and communicating these initiatives; and (iii) bring together in one place the most recent information on elimination and eradication goals and timelines, their associated targets, and assessment processes (formal processes to document the achievement of a goal in a country or region, led by WHO) for the infectious conditions targeted by these goals.

## Methods

We conducted this review and report its findings according to the Preferred Reporting Items for Systematic Reviews and Meta-Analyses (PRISMA) reporting standards.^13^ The study is registered in PROSPERO (CRD42018099733).

### Search strategies

We searched WHO’s Institutional Repository for Information Sharing for documents that were authored or co-authored by WHO, published between 2008 and 2022, in any language, and with titles containing: “elimination” OR “eliminating” OR “eliminate” AND/OR “eradication” OR “eradicating” OR “eradicate”. In addition, we conducted a Google Advanced Search using the same terms (not restricted to titles only), limited to the WHO domain (http://www.who.int) and in the six official WHO languages – Arabic, Chinese, English, French, Russian and Spanish. We used these two cluster terms separately for elimination and eradication documents to make sure that we included documents with either or both terms. We then combined the searches. We conducted the searches on 2 and 3 August 2022 for elimination and eradication, respectively.

We also contacted WHO headquarters to identify other documents for possible inclusion. Lastly, we checked reference lists of the fully reviewed records to identify publications on the infectious conditions whose latest strategies were published before 2008, and documents that contained universally accepted targets for an infection but were authored by other organizations, for example, the Joint United Nations Programme on HIV/AIDS (UNAIDS). We did not put any restrictions on publication date for documents retrieved through the last two strategies. We did not search bibliographic databases such as PubMed® and Embase® because goals related to infectious disease elimination and eradication are defined by WHO and its partner organizations, and the relevant documents are published on these organizations’ websites rather than in peer-reviewed journal articles.

### Eligibility criteria

Inclusion criteria were: documents that comprehensively described elimination or eradication strategies, including goals, timelines, targets and assessment processes for infectious conditions at the global level or at a specific geographical level, such as the WHO regions.

Exclusion criteria were: (i) documents on non-infectious conditions; (ii) documents on infectious conditions that were educational or media material, older progress reports (global and/or regional) and technical documents on treatment, vaccines and survey methods; (iii) country-specific reports – except for neglected tropical diseases where the infection is predominantly confined to that country; and (iv) documents on infectious conditions targeted for global disease control only.

Where more than one report described the goals, targets or assessment processes, we only included the most recent document. Where two or more reports described non-overlapping components (for example, goals versus assessment process, or a strategical transition from one goal to another), we included all documents.

### Search and review process

**Fig. 1:**
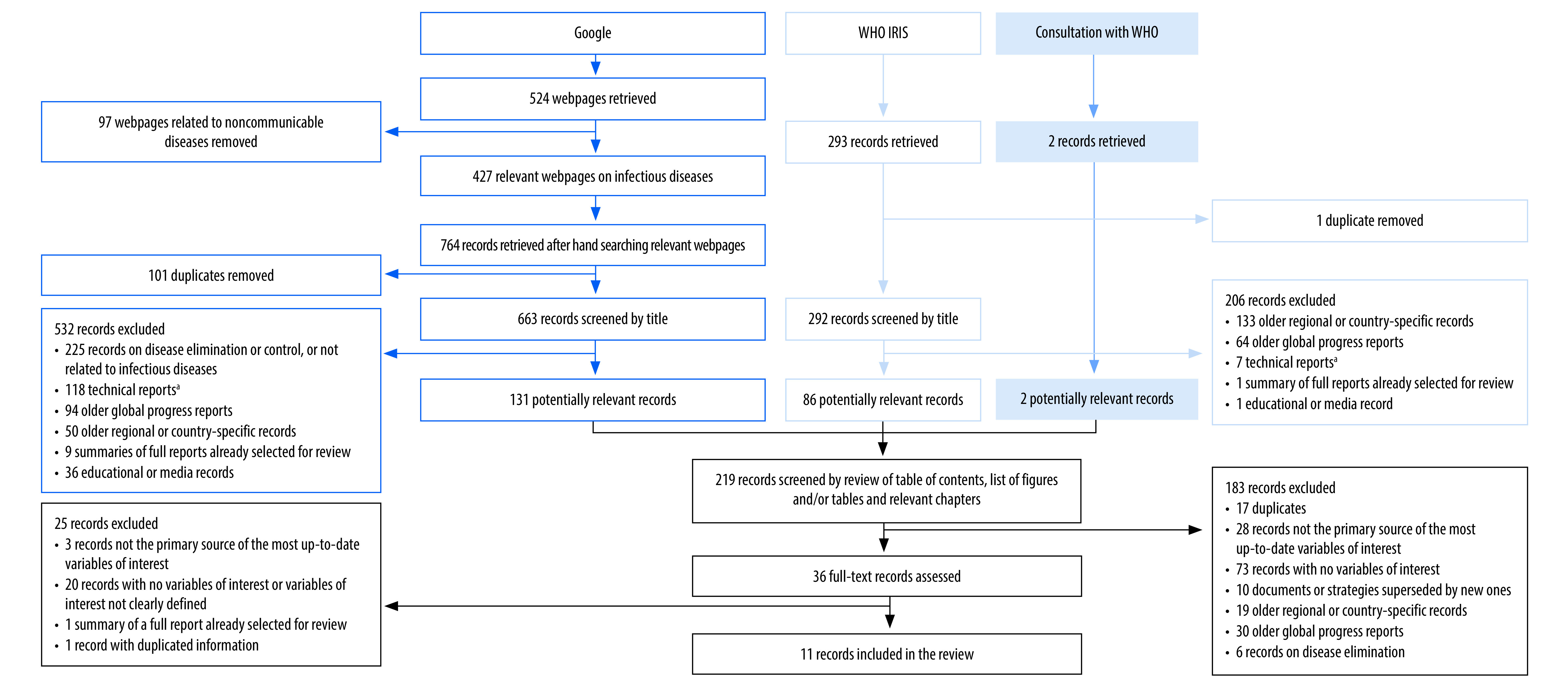
Flow diagram of selection of documents on disease eradication

IRIS: Institutional Repository for Information Sharing; WHO: World Health Organization.

^a^ Technical reports were operational and meeting reports related to pesticides, vectors, vaccines, testing and treatment as well as survey methods.

**Fig. 2:**
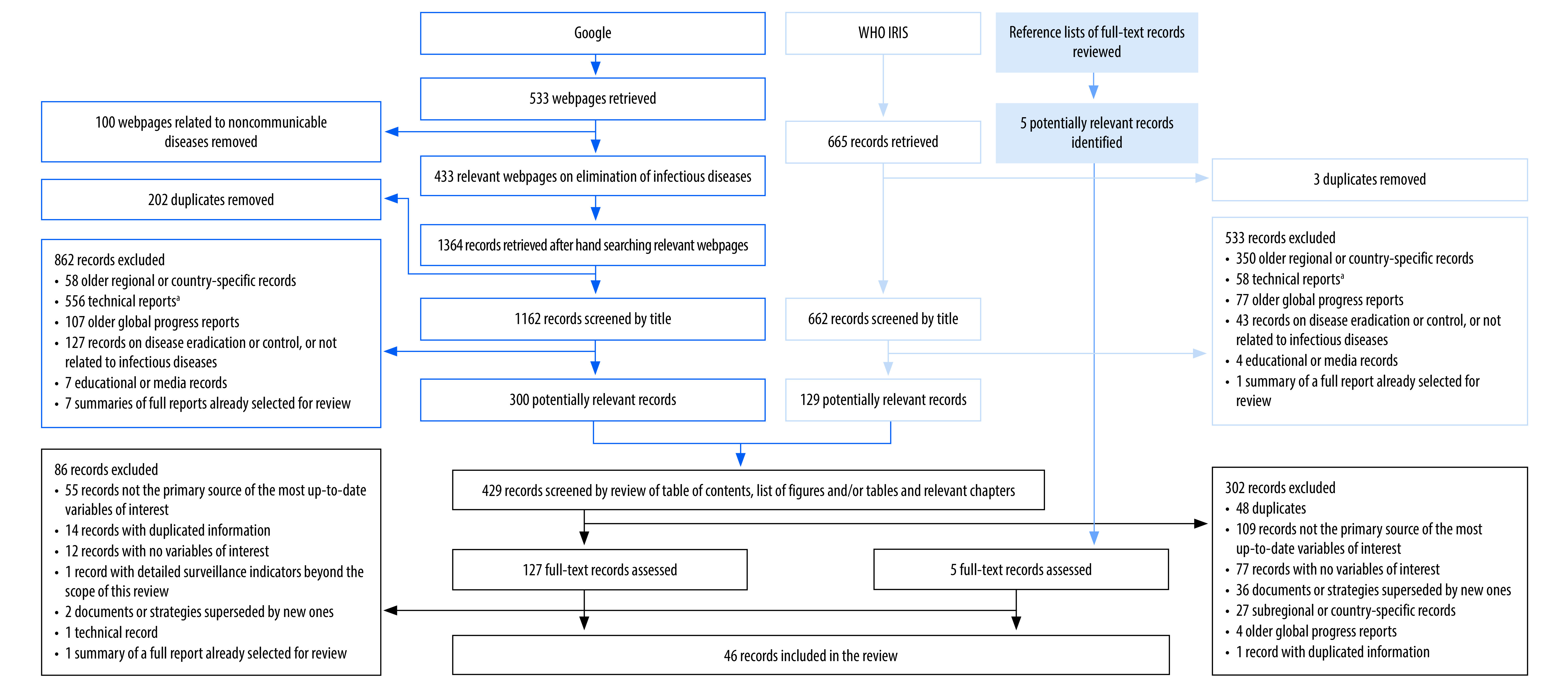
Flow diagram of selection of documents on disease elimination

IRIS: Institutional Repository for Information Sharing; WHO: World Health Organization.

^a^ Technical reports were operational and meeting reports related to pesticides, vectors, vaccines, testing and treatment as well as survey methods.

Two researchers searched for documents and screened titles, table of contents and foreword for elimination and eradication. The researchers then independently screened full-text documents to assess eligibility (Fig. 1 and Fig. 2). Disagreements were discussed with a third researcher and resolved by consensus.

### Data extraction and analysis

A standardized data extraction tool captured information on the variables of interest. We stored the extracted data in an Excel file (Microsoft, Redmond, United States of America). 

We extracted data on the following variables: (i) infection type and name of the infectious condition; (ii) goals and timelines to achieve the goals; (iii) definitions of the goals; (iv) assessment process; (v) epidemiological endpoints and their impact targets – measures such as prevalence or incidence that are used to measure the impact of an intervention; (vi) interventions and their process targets – interventions being the public health responses to address the disease of interest, and process targets being the benchmarks to be achieved for these interventions; and (vii) other, such as the geographical focus of the goal, achievement of goal, and the different case definitions important to understand the goals, for example, imported versus endemic cases.

## Results

The search strategies retrieved 219 documents on eradication and 429 on elimination. Of these documents, we selected 36 on eradication and 127 on elimination for full text review. After review of the references, we included an additional five documents on elimination. After full text review, we included 11 eradication and 46 elimination documents in the review. Two documents included information on both eradication and elimination and were included in both categories; therefore, 55 documents met the relevant eligibility criteria (Fig. 1; Fig. 2 and Table 1).

**Table 1:** Documents included in the systematic review on elimination and eradication goals and targets for communicable diseases

Author	Year published		Disease focus
**Eradication documents**			
WHO^14^	1967		Smallpox
Fenner, et al.^15^	1988		Smallpox
WHO^16^	2012		Neglected tropical diseases
WHO^17^	2012		Yaws
Global Polio Eradication Initiative, WHO, CDC and UNICEF^18^	2013		Polio
WHO^19^	2017		Neglected tropical diseases
WHO^20^	2018		Yaws
WHO^21^	2018		Yaws
WHO^22^	2020		Neglected tropical diseases
WHO^23^	2021		Yaws
Global Polio Eradication Initiative, WHO, Rotary International, CDC, UNICEF and Bill and Melinda Gates Foundation^24^	2021		Polio
**Elimination documents**			
WHO^25^	2011	Lymphatic filariasis	
WHO^26^	2011	Schistosomiasis	
WHO^16^	2012	Neglected tropical diseases	
WHO^27^	2014	Tuberculosis	
WHO Regional Office for Europe^28^	2014	Measles and rubella	
WHO^29^	2015	Malaria	
WHO^30^	2015	Tuberculosis	
WHO^31^	2015	Tuberculosis	
WHO^32^	2016	Human onchocerciasis	
WHO^33^	2016	Trachoma	
WHO Regional Office for South-East Asia^34^	2016	Kala-azar	
WHO^35^	2017	Cholera	
WHO^36^	2017	Lymphatic filariasis	
WHO Regional Office for the Western Pacific^37^	2017	Measles, rubella and congenital rubella syndrome	
Pan American Health Organization; WHO Regional Office for the Americas^38^	2017	Measles, rubella and congenital rubella syndrome	
Pan American Health Organization; WHO Regional Office for the Americas^39^	2017	Mother-to-child transmission of HIV, syphilis, hepatitis B and Chagas disease	
WHO, FAO and World Organization for Animal Health^40^	2018	Rabies	
WHO^41^	2018	Rabies	
WHO, UNICEF, Gavi^42^	2018	Yellow fever epidemics	
WHO Regional Office for the Western Pacific^43^	2018	Mother-to-child transmission of HIV, hepatitis B and syphilis	
WHO^44^	2019	Tetanus	
WHO Regional Office for South-East Asia^45^	2019	Measles,rubella and congenital rubella syndrome	
WHO^46^	2019	Soil-transmitted helminthiases	
WHO^47^	2020	Cervical cancer	
WHO^22^	2020	Neglected tropical diseases	
WHO^48^	2020	Leprosy	
WHO^49^	2020	Malaria	
WHO^50^	2020	Measles, rubella and congenital rubella syndrome	
WHO Regional Office for South-East Asia^51^	2020	Measles, rubella and congenital rubella syndrome	
WHO Regional Office for Europe^52^	2020	Tuberculosis	
WHO^53^	2021	Trachoma	
WHO Regional Office for South-East Asia^54^	2021	Cervical cancer	
WHO Regional Office for South-East Asia^55^	2021	Leprosy	
WHO Regional Office for South-East Asia^56^	2021	Leprosy	
WHO^57^	2021	Lymphatic filariasis	
WHO^58^	2021	Malaria	
WHO Regional Office for the Eastern Mediterranean^59^	2021	Measles and rubella	
Pan American Health Organization; WHO Regional Office for the Americas^60^	2021	Measles, rubella, and congenital rubella syndrome	
WHO^61^	2021	Meningitis	
WHO^62^	2021	Mother-to-child transmission of HIV, syphilis and hepatitis B virus	
WHO^63^	2021	Viral hepatitis	
WHO^64^	2022	HIV, viral hepatitis and sexually transmitted infections	
WHO^65^	2022	Malaria	
WHO^66^	2022	Maternal and neonatal tetanus	
WHO^67^	2022	Human schistosomiasis	
WHO^68^	2022	Human African trypanosomiasis (gambiense and rhodesiense)	

CDC: Centers for Disease Control and Prevention; FAO: Food and Agriculture Organization of the United Nations; Gavi: Gavi, the Vaccine Alliance; UNICEF: United Nations Children’s Fund; WHO: World Health Organization.

### Types of goals

We identified eight goals related to some form of elimination or eradication (Table 2). These goals were defined for 27 infectious conditions (available in the online repository).^69^ A goal of disease control was also defined for three infectious conditions, giving a total of nine goals. The goal categories were not mutually exclusive across infectious conditions – 11 conditions had more than one goal (Chagas disease, cholera, human African trypanosomiasis (gambiense), human immunodeficiency virus (HIV), leishmaniasis, leprosy, rabies, schistosomiasis, syphilis, tuberculosis and viral hepatitis B; Table 2). Only smallpox had met the goal of worldwide permanent reduction to zero and was certified as eradicated in 1980. Leprosy alone had met the global goal of elimination as a public health problem (in 2000), and human African trypanosomiasis (gambiense) had partially met that goal (in 2020).

**Table 2:** Types of goals and infectious conditions, their timelines and assessment processes

Goal type, by infection type and infectious condition	Scope	Target date	Year achieved^a^	Assessment process and geographical level of award
**Worldwide permanent reduction to zero**				
Bacterial				
Yaws^16^^,^^17^^,^^20^^–^^23^	Global	2030	NA	Verification: country level; certification: global level
Parasitic				
Dracunculiasis^16^^,^^19^^,^^22^	Global	2030	NA	Certification: country & global levels
Viral				
Polio^18^^,^^24^	Global	2026	NA	Certification: regional and global levels
Smallpox^14^^,^^15^	Global	10 years from 1966	1977	Certification: continental and global levels
**Interruption of endemic transmission^b^**				
Bacterial				
Cholera^c,^^35^	Global (20 of 47 endemic countries)	2030	NA	Process not yet defined
Leprosy^c,^^22^^,^^48^^,^^55^^,^^56^	Global	2030	NA	Verification: country level
Parasitic				
Malaria^29^^,^^49^^,^^58^^,^^65^	Global	2030	NA	Certification: country level; verification: subnational level
Viral				
Measles^28^^,^^37^^,^^38^^,^^45^^,^^50^^,^^51^^,^^59^^,^^60^	Global	Varies by WHO region^d^	See note^d^	Verification: country and/or regional level
Rubella and congenital rubella syndrome^28^^,^^37^^,^^38^^,^^45^^,^^50^^,^^51^^,^^59^^,^^60^	Global	Varies by region^d^	See note^d^	Verification: country and/or regional level
**Interruption of transmission**				
Parasitic				
Human African trypanosomiasis (gambiense)^c,^^22^^,^^68^	Global	2030	NA	Verification: country level
Onchocerciasis^22^^,^^32^	African, Americas and Eastern Mediterranean regions	2030	NA	Verification: country level
Schistosomiasis^c,^^22^^,^^67^	Global (25 of 78 endemic countries)	2030	NA	Verification: country level
Viral				
Rabies^c,^^40^^,^^41^	Global	No date	NA	Verification: country level
**Elimination as a public health problem**				
Bacterial				
Leprosy^22^^,^^48^^,^^55^^,^^56^	Global	2000	2000^f^	Validation: country level^e^
Maternal and neonatal tetanus^44^^,^^66^	Global	2020	Not fully achieved^f^	Validation: country level
Trachoma^22^^,^^33^^,^^53^	Global	2030	NA	Validation: country level
Tuberculosis^c,^^27^^,^^31^^,^^52^	North America, western Europe and western Pacific (low-incidence countries)	2050	NA	Not mentioned
Parasitic				
Chagas disease^c,^^22^	Americas, European and Western Pacific regions	2030	NA	Verification: country level^g^
Human African trypanosomiasis (gambiense)^22^^,^^68^	Global	2020	One of two impact targets was met in 2020^h^	Validation: country level
Lymphatic filariasis^22^^,^^25^^,^^36^^,^^57^	Global	2030	NA	Validation: country level
Human African trypanosomiasis (rhodesiense)^22^^,^^68^	East Africa	2030	NA	Validation: country level
Schistosomiasis^22^^,^^67^	Global (all 78 endemic countries)	2030	NA	Validation: country level
Soil-transmitted helminths^22^^,^^46^	Global	2030	NA	Validation: geographic level not mentioned
Visceral leishmaniasis^c,^^22^^,^^34^	Global	2030	NA	Validation: country level
Viral				
Rabies^22^^,^^40^^,^^41^	Global	2030	NA	Validation: country level
Human papillomavirus-related cervical cancer^47^^,^^54^	Global	End of century	NA	Not mentioned
**Elimination of vertical transmission as a public health problem**				
Bacterial				
Syphilis^c,^^39^^,^^43^^,^^62^^,^^64^	Global	2030	NA	Validation: country level
Parasitic				
Chagas disease^22^^,^^39^	Region of the Americas	2020	Not achieved^h^	Verification: country level
Viral				
HIV^c,^^39^^,^^43^^,^^62^^,^^64^	Global	2030	NA	Validation: country level
Hepatitis B virus^c,^^39^^,^^43^^,^^62^^,^^64^	Global	2030	NA	Validation: country level
**Elimination as a public health threat**				
Viral				
Viral hepatitis B and C^63^^,^^64^	Global	2030	NA	Validation: country level
**Pre-elimination**				
Bacterial				
Tuberculosis^27^^,^^52^	North America, western Europe and western Pacific (low-incidence countries)	2035	NA	Not mentioned
**End of disease epidemic**				
Bacterial				
Cholera^35^	47 countries affected by cholera	2030	NA	Not mentioned
Meningitis A^i,^^61^	Global	2030	NA	Not mentioned
STIs^j,23^	Global	2030	NA	Not mentioned
Tuberculosis^30^^,^^31^	Global	2035	NA	Not mentioned
Viral				
HIV/AIDS^k,^^64^	Global	2030	NA	Not mentioned
Yellow fever^l,^^42^	Global	2026	NA	Not mentioned
**Disease control**				
Parasitic				
Chagas disease (oral transmission)^16^	Region of the Americas	No date	No update	None
Cutaneous leishmaniasis^22^	Global	2030	NA	None
Schistosomiasis^26^	52 of 78 endemic countries)	2020	No update	None

AIDS: acquired immunodeficiency syndrome; HIV: human immunodeficiency virus; NA: not applicable; STIs: sexually transmitted infections; WHO: World Health Organization.

^a^ Where the target date is 2030 and not yet reached, we have put not applicable; however, some individual countries could have met these targets ahead of their timeline.

^b^ Interruption of endemic transmission is defined by the ability of surveillance systems to identify local versus imported cases (online repository).^69^

^c^ Infectious conditions with more than one goal.

^d^ European, 2015; Americas, 2023; Western Pacific, 2020; and South-East Asia, 2023. An update on the status of the 2015 and 2020 targets (achieved or not) was not provided in the documents reviewed.

^e^ In case of elimination of leprosy as a public health problem: the target has been met by all countries, except Brazil; although no formal validation process was developed as the target indicator could be determined through a straightforward mathematical calculation.

^f^ 12 of 59 priority countries are yet to achieve the goal, while 47 have been validated as having met the goal.

^g^ Although the goal for Chagas disease is elimination as a public health problem, this goal is achieved by interruption of transmission through four out of six transmission routes – vector, blood transfusion, organ transplantation and congenital; the other two routes are food and laboratory accidents. Therefore, the assessment process is verification of interruption of transmission through these four routes, and not validation.

^h^ By 2020, 0/41 countries had achieved the goal of elimination of vertical transmission of Chagas disease. An update on the status of the disease control target via oral transmission (achieved or not) was not provided in the documents reviewed.

^i^ For meningitis, the goal is to eliminate vaccine-preventable bacterial meningitis epidemics as a public health threat.

^j^ Primarily bacterial STIs, gonorrhoea (bacterial), syphilis (bacterial) and chlamydia (bacterial). However, one overall target also includes trichomoniasis (parasitic). For STIs, the goal is to end the STI epidemic as a public health concern.

^k^ The goal is to end the AIDS/HIV epidemic as a public health threat.

^l^ The goal is to eliminate yellow fever epidemics.

### Definitions

The documents related to the goals of worldwide permanent reduction to zero, and interruption of transmission or endemic transmission, had definitions of goals that matched their respective definitions of eradication and elimination of disease or infection shown in Box 1. Elimination as a public health problem, a goal related to both infection and disease, was defined in eight documents reviewed as achievement of the measurable targets set by WHO, which when reached required continued action to maintain the targets.^22^^,^^36^^,^^44^^,^^47^^,^^55^^,^^56^^,^^62^^,^^63^ All documents with goals for elimination as a public health problem had measurable impact targets for specific diseases that coincided with the definition of the goal, and matched the definition of disease control shown in Box 1. One document defined the goal of elimination as a public health threat as equivalent to the goal of elimination as a public health problem.^63^ The documents with goals of pre-elimination and end of disease epidemic had measurable impact targets for different levels of reduction in disease prevalence, incidence, morbidity or mortality. These targets coincided with the definition of disease control in Box 1. For the goal of end of disease epidemic, most conditions had percentage reduction thresholds for impact targets defined at a global level, with countries being encouraged to develop appropriate targets for the local context. In comparison, for different subclassifications of a public health-related goal (elimination as a public health problem or threat, elimination of vertical transmission and pre-elimination), all conditions had impact targets based on case numbers, rates or prevalence percentage defined at a national or subnational level (Table 3).

**Table 3:** Disease endpoints and thresholds, by goal type and infectious condition

Goal type, by infectious condition	Disease endpoint	No. of thresholds	Type of threshold		
			Target	Rate	% reduction or fractional reduction
**Worldwide permanent reduction to zero**					
Dracunculiasis^16^^,^^19^^,^^22^	Cases	1	Zero	NR	NR
Polio^18^^,^^24^	Cases	1	Zero	NR	NR
Smallpox^14^^,^^15^	Cases	1	Zero	NR	NR
Yaws^16^^,^^17^^,^^20^^–^^23^	Cases	1	Zero	NR	NR
**Interruption of endemic transmission**					
Cholera^a,21^	Case	1	Zero, endemic, nationally	NR	NR
Leprosy^22^^,^^48^^,^^55^^,^^56^	New cases	4	Zero, new autochthonous cases, nationally. 62 500 new cases, globally	0.12/1 000 000 new cases with grade 2 disabilities, globally	90% reduction in new case rate in children, globally
Malaria^29^^,^^49^^,^^58^^,^^65^	Incidence	2	Zero indigenous cases, nationally	NR	90% reduction by 2030, globally
	Mortality	1	NR	NR	90% reduction by 2030, globally
Measles^28^^,^^37^^,^^38^^,^^45^^,^^50^^,^^51^^,^^59^^,^^60^	Cases	1	Zero, endemic, regionally	NR	NR
Rubella and congenital rubella syndrome ^28^^,^^37^^,^^38^^,^^45^^,^^50^^,^^51^^,^^59^^,^^60^	Cases	1	Zero, endemic, regionally	NR	NR
**Interruption of transmission**					
Human African trypanosomiasis (gambiense)^22^^,^^68^	Cases	1	Zero, nationally	NR	NR
Onchocerciasis^22^^,^^32^	Incidence	1	NR	Zero, nationally	NR
Rabies^40^^,^^41^	Cases in dogs	1	Zero canine cases, nationally	NR	NR
Schistosomiasis^22^^,^^67^	Incidence	1	NR	Zero	NR
**Elimination as a public health problem**					
Chagas disease^22^	Incidence	1	Zero,^b^ nationally	NR	NR
Human African trypanosomiasis (gambiense)^22^^,^^68^	Cases	2	< 2000 a year, globally	< 1/10 000 a year (in at-risk areas)	NR
Leprosy^22^^,^^48^^,^^55^^,^^56^	Prevalence	1	NR	< 1 case/10 000, nationally	NR
Lymphatic filariasis^22^^,^^25^^,^^36^^,^^57^	Prevalence	3	< 2% antigenaemia in all endemic areas^c^	NR	NR
			< 1% antigenaemia in all endemic areas^d^	NR	NR
			< 2% antibody prevalence in all endemic areas, nationally^e^	NR	NR
Maternal and neonatal tetanus^44^^,^^66^	Incidence	1	NR	< 1/1000 live births a year per district	NR
Rabies^22^^,^^40^^,^^41^	Mortality	1	Zero human deaths, nationally	NR	NR
Human African trypanosomiasis (rhodesiense)^22^^,^^68^	Cases	1	NR	< 1/10 000 a year per district	NR
Schistosomiasis^22^^,^^67^	Prevalence	1	< 1% of heavy-intensity infections, nationally^f^	NR	NR
Soil-transmitted helminths^22^^,^^46^	Prevalence	1	< 2% of moderate-to-heavy intensity infections in pre-school and school-aged children, nationally^g^	NR	NR
Trachoma^22^^,^^33^^,^^53^	Prevalence	2	< 0.2% TT in ≥ 15-year-olds, nationally	NR	NR
			< 5% TF in children, nationally	NR	NR
Tuberculosis (low-incidence countries)^h,^^27^^,^^31^^,^^52^	Incidence	1	NR	< 1 case/1 000 000, nationally	NR
Viral STIs – human papillomavirus-related cervical cancer^47^^,^^54^	Incidence	2	NR	4 cases/100 000 women-years, nationally	South-East Asian Region: reduce by one third by 2030
	Mortality	1	NR	NR	South-East Asian Region: reduce by one third by 2030
Visceral leishmaniasis^22^^,^^34^	Cases	1	NR	South-East Asian Region: < 1 case/10 000	NR
	Case fatality	1	For all countries other than in South-East Asian Region: < 1%	NR	NR
**Elimination of vertical transmission as a public health problem**					
Chagas^22^^,^^39^	Transmission rate	1	Zero, nationally	NR	NR
	Prevalence	1	≥ 90% children cured, nationally	NR	NR
Hepatitis B virus^39^^,^^43^^,^^62^^,^^64^	Prevalence	1	≤ 0.1% HBsAG prevalence in children < 5 years, nationally	NR	NR
	Transmission rate	1	< 2%, nationally^i^	NR	NR
HIV^39^^,^^43^^,^^62^^,^^64^	New cases	1	NR	≤ 50/100 000 live births, nationally	NR
	Transmission rate	1	< 5% and < 2% in breastfeeding and non-breastfeeding countries, respectively^j^	NR	NR
Syphilis^39^^,^^43^^,^^62^^,^^64^	New cases	1	NR	≤ 50/100 000 live births, nationally	NR
**Elimination as a public health threat**					
Viral hepatitis B (national level targets)^63^^,^^64^	Prevalence	2	0.5% HBsAg prevalence in children 0–5 years by 2025^k^	NR	NR
			0.1% HBsAg prevalence in children 0–5 years by 2030^k^	NR	Or 95% reduction by 2030^l^
	Incidence	2	NR	11/100 000 cases a year by 2025	NR
				2/100 000 cases a year by 2030	NR
	Mortality	2	NR	7/100 000 deaths a year by 2025	NR
				4/100 000 deaths a year by 2030	Or 65% reduction by 2030^l^
Viral hepatitis C (national level targets)^63^^,^^64^	Incidence	4	NR	13/100 000 cases a year by 2025	NR
				5/100 000 cases a year by 2030	Or 80% reduction by 2030^l^
				People who inject drugs: 3/100 a year by 2025	NR
				People who inject drugs: 2/100 a year by 2030	NR
	Mortality	2	NR	3/100 000 deaths a year by 2025	NR
				2/100 000 deaths a year by 2030	Or 65% reduction by 2030^l^
**Pre-elimination**					
Tuberculosis (low-incidence countries)^27^^,^^52^	Incidence	1	NR	< 10/1 000 000 cases by 2035, nationally	Or 90% reduction by 2035^l^
**End of disease epidemic**					
Cholera^35^	Mortality	1	9500 deaths by 2030	NR	Or 90% reduction by 2030, globally
	Outbreaks	1	Zero, uncontrolled	NR	NR
Meningitis A^61^	New cases	1	NR	NR	50% reduction by 2030, globally
	Mortality	1	NR	NR	70% reduction by 2030, globally
HIV/AIDS^64^	New cases	6	All ages: 370 000/year by 2025, globally	0.05/1000 uninfected population a year	Or 75% reduction, globally
			All ages: 335 000/year by 2030, globally	0.025/1000 uninfected population a year	Or 78% reduction, globally^m^
			0–14 years: 20 000/year by 2025, globally	NR	Or 86% reduction, globally
			0–14 years: 15 000/year by 2030, globally	NR	Or 90% reduction, globally^m^
	Mortality	2	250 000 deaths/year by 2025, globally	NR	Or 63% reduction, globally
			< 240 000 deaths/year by 2030, globally	NR	Or >65% reduction, globally
	Mortality from comorbidity	2	110 000 deaths/year by 2025, globally^n^	NR	Or 48% reduction, globally
			55 000 deaths/year by 2030, globally^n^	NR	Or 74% reduction, globally
STIs (bacterial)^64^					
Syphilis	New cases	2	5 700 000/year by 2025, globally	NR	Or 20% reduction, globally
			710 000/year by 2030, globally	NR	Or 90% reduction, globally^m^
Gonorrhoea	New cases	2	65 800 000/year by 2025, globally	NR	Or 20% reduction, globally
			8 230 000/year by 2030, globally	NR	Or 90% reduction, globally^m^
STIs (overall): chlamydia, gonorrhoea, syphilis, trichomoniasis^64^	New cases	2	< 300 000 000/year by 2025, globally	NR	Or 20% reduction, globally
			< 150 000 000/year by 2030, globally	NR	Or 60% reduction, globally
Tuberculosis (high-incidence countries)^o,^^30^^,^^31^	Incidence	2	NR	< 20/100 000 cases by 2030, nationally	Or 80% reduction, globally^l^
			NR	< 10/100 000 cases by 2035, nationally	Or 90% reduction, globally^l^
	Mortality	2	NR	NR	90% reduction by 2030, globally
					95% reduction by 2035, globally
Yellow fever^42^	Outbreaks	1	Zero, uncontrolled	NR	NR
**Disease control**					
Chagas disease (oral route)^16^	NR	NA	NR	NR	NR
Cutaneous leishmaniasis^22^	No impact targets for disease endpoint	NA	NR	NR	NR
Schistosomiasis^26^	Prevalence	1	< 5% heavy-intensity infections^f^	NR	NR

AIDS: acquired immunodeficiency syndrome; HBsAG: hepatitis B surface antigen; HIV: human immunodeficiency virus; NA: not applicable; NR: not reported; STIs: sexually transmitted infections; TF: trachomatous inflammation – follicular; TT: trachomatous trichiasis.

^a^ The target is for 20 endemic countries to eliminate cholera.

^b^ Based on four of six transmission routes, that is, vectoral, transfusion, transplantation and congenital. The other two transmission routes are oral and laboratory accidents.

^c^ In areas where *Wuchereria bancrofti* is endemic and *Anopheles* or *Culex* is the main vector.

^d^ In areas where *Aedes* is the main vector.

^e^ In areas where *Brugia* spp. is endemic.

^f^ Heavy intensity of infections: *Schistosoma mansoni* (400 eggs/g faeces, *S*. *haematobium* (50 eggs/10 mL urine).

^g^ Caused by *Ascaris lumbricoides*, *Trichuris trichiura*, *Necator americanus* and *Ancylostoma duodenale.*

^h^ For details on the impact targets for the European region, see online repository.^69^

^i^ Additional target for countries using targeted timely hepatitis B vaccine birth dose.

^j^ Breastfeeding countries: countries where the benefits of breastfeeding in terms of child survival outweigh the risk of HIV transmission via breastfeeding. Non-breastfeeding countries: countries where women living with HIV who give birth are strongly recommended to avoid breastfeeding due to evidence of a risk of HIV transmission via breastfeeding.

^k^ Childhood prevalence is a proxy for incidence of chronic hepatitis B virus infection.

^l^ For viral hepatitis, the relative reduction targets are from a 2015 baseline; while the absolute targets take the baseline of 2020, however, the relative reduction targets from a 2020 baseline can be calculated from document ref# 64; likewise, for TB, the relative reduction targets are from a 2015 baseline.

^m^ From a 2020 baseline.

^n^ Mortality associated with causes related to tuberculosis, hepatitis B and C.

^o^ The targets can be adapted nationally depending on the baseline point.

Note: For shared targets with HIV, viral hepatitis and STIs, see online repository.^69^

The sequential nature of the goals for cholera, human African trypanosomiasis (gambiense), leprosy, rabies, schistosomiasis and tuberculosis, and their varying thresholds, indicated the progression of goals on the disease-control spectrum (Table 2 and Table 3). For instance, tuberculosis goals ranged from ending the epidemic in high-incidence countries (defined as 90% reduction in incidence, equivalent to 10 cases per 100 000 population by 2035); to pre-elimination in low-incidence countries (defined as 90% reduction in incidence, equivalent to < 10 cases per 1 000 000 population by 2035); and elimination as a public health problem in low-incidence countries (defined as < 1 case per 1 000 000 population by 2050; Table 3). See online repository for quantitative impact targets.^69^

### Non-standardized terms

Some non-standardized terms were used to describe certain goals. These terms included: (i) *virtual* elimination for vertical transmission of HIV as a public health problem at < 2% and < 5% transmission rate in breastfeeding and non-breastfeeding countries, respectively;^62^ (ii) public health *threat* for cholera, meningitis A, HIV and viral hepatitis B and hepatitis C;^35^^,^^61^^,^^63^^,^^64^ and (iii) public health *concern* for bacterial sexually transmitted infections.^64^ Standardized definitions for a condition deemed to be a threat or concern were not provided. Some potentially misleading terms were used for the goal of end of disease epidemic; for example, *eliminate* disease epidemics was used for yellow fever and meningitis A.^42^^,^^61^

### Interventions and process targets

We broadly categorized interventions into five groups: prevention, early detection, clinical management, surveillance and other, and presented the quantitative process targets (online repository).^69^

### Assessment processes

Certification was the main assessment process for the goal of worldwide permanent reduction to zero, while verification was the main process for the goals of interruption of transmission and endemic transmission. Validation was the main process for the goals of elimination as a public health problem or threat, and elimination of vertical transmission. One infectious condition (Chagas disease) with a goal of elimination as a public health problem had verification as an assessment process, because the elimination strategy involves interruption of transmission through four of six transmission routes, while disease control is the goal for the remaining two routes. We did not identify any assessment processes for the goals of pre-elimination, end of disease epidemic and disease control.

## Discussion

This systematic review investigated elimination and eradication goals for infectious conditions, and their associated definitions, terminology, targets and assessment processes. We identified nine different goals, ranging from disease control to eradication across 27 infectious conditions. These goals were not mutually exclusive for these conditions and 11 conditions had more than one goal. Goals had been met for only two conditions. This review highlights the progression of goals along a disease-control continuum, such as end of disease epidemic to pre-elimination to elimination as a public health problem or threat. A clear understanding of where the specific infectious disease goals fall on this continuum of disease control is important to avoid misperceptions and miscommunication of overall objectives. As we approach the 2030 target date to achieve many of these goals, and for other infections to become candidates for elimination in the future, clarity of definitions and objectives is important for public health professionals and policy-makers.

We found that a range of terms have been used to classify infectious conditions, including a public health concern, problem and threat. Criteria exist for classification of an infectious condition as a public health problem, namely: (i) high burden of disease and a likely increasing trend; (ii) large burden in terms of morbidity and/or mortality, quality of life and cost; and (iii) a feasibility to take action on the condition at the community or public health level.^70^^–^^72^ The use of non-standardized terminology that we identified makes it unclear how and when a condition is deemed a public health threat or a concern based on these three criteria. We therefore propose standardized definitions for the terms public health threat and public health concern (Box 2). For meningitis A and yellow fever, the term eliminate disease epidemics was used,^42^^,^^61^ which may cause confusion as to whether the goal is to end the disease epidemic or eliminate the disease as a public health problem or threat. We recommend that WHO considers standardizing terminology across all infectious conditions targeted for elimination or eradication.

Box 2 **Proposed definitions of public health concern and public health threat for infectious diseases**

**Public health concern**

A public health concern is an infectious condition that affects a significant proportion of a specific population and fulfils the following criteria: (i) likely increasing in trend or has a potential for outbreaks and/or community spread; (ii) high burden in terms of morbidity and quality of life; (iii) low overall risk of death; (iv) perceived as low-to-moderate risk by the general public; and (v) feasible to act on the condition at a community level.

**Public health threat**

A public health threat is an infectious condition or problem that potentially affects a significant proportion of a specific population and fulfils the following criteria: (i) likely increasing in trend or has a potential for outbreaks and/or community spread; (ii) high burden in terms of morbidity and/or mortality and quality of life; (iii) high overall risk of death; (iv) perceived as high risk by the general public; and (v) feasible to act on the condition at a community level.

Noteworthy is that for the goal of end of disease epidemic, with relative reduction global targets, a so-called one-size-fits-all approach is not appropriate, as these targets could mean different things to different countries depending upon their endemicity and starting point. WHO encourages countries to adapt strategic directions and goals to local epidemiological and health system contexts.^64^ Some countries therefore have locally adapted targets for infectious conditions, such as HIV/AIDS. However, discrepancies may exist between the selected goal and its targets. For example, England has a goal of “ending HIV transmission” or “to eradicate HIV transmission”^10^^,^^11^ with a target of < 100 new cases a year by 2030.^73^ Australia has a goal of “ending HIV transmission” or “virtual elimination of HIV transmission,” with a target of 90% reduction in HIV infections by 2025 compared with 2010.^9^ Ending transmission of HIV is not the same as ending the HIV epidemic; the former implies interrupting transmission (zero cases), which is a misnomer if the target threshold is greater than zero. Likewise, we found that WHO only used the term virtual elimination for elimination of vertical transmission of HIV as a public health problem. Importantly, this term cannot be used interchangeably with ending or interrupting transmission. Clarity about these definitions is crucial so that important programmes are not prematurely de-funded. We recommend that countries aiming for a measurable elimination target that has a threshold of greater than zero cases consider aligning a more suitable goal to this threshold, such as elimination of HIV as a public health problem or threat. We also urge the scientific and health policy communities not to use the term eradication for a limited geographical location, as this term is reserved for worldwide interruption of transmission, where intervention measures are no longer needed anywhere.

**Fig. 3:**
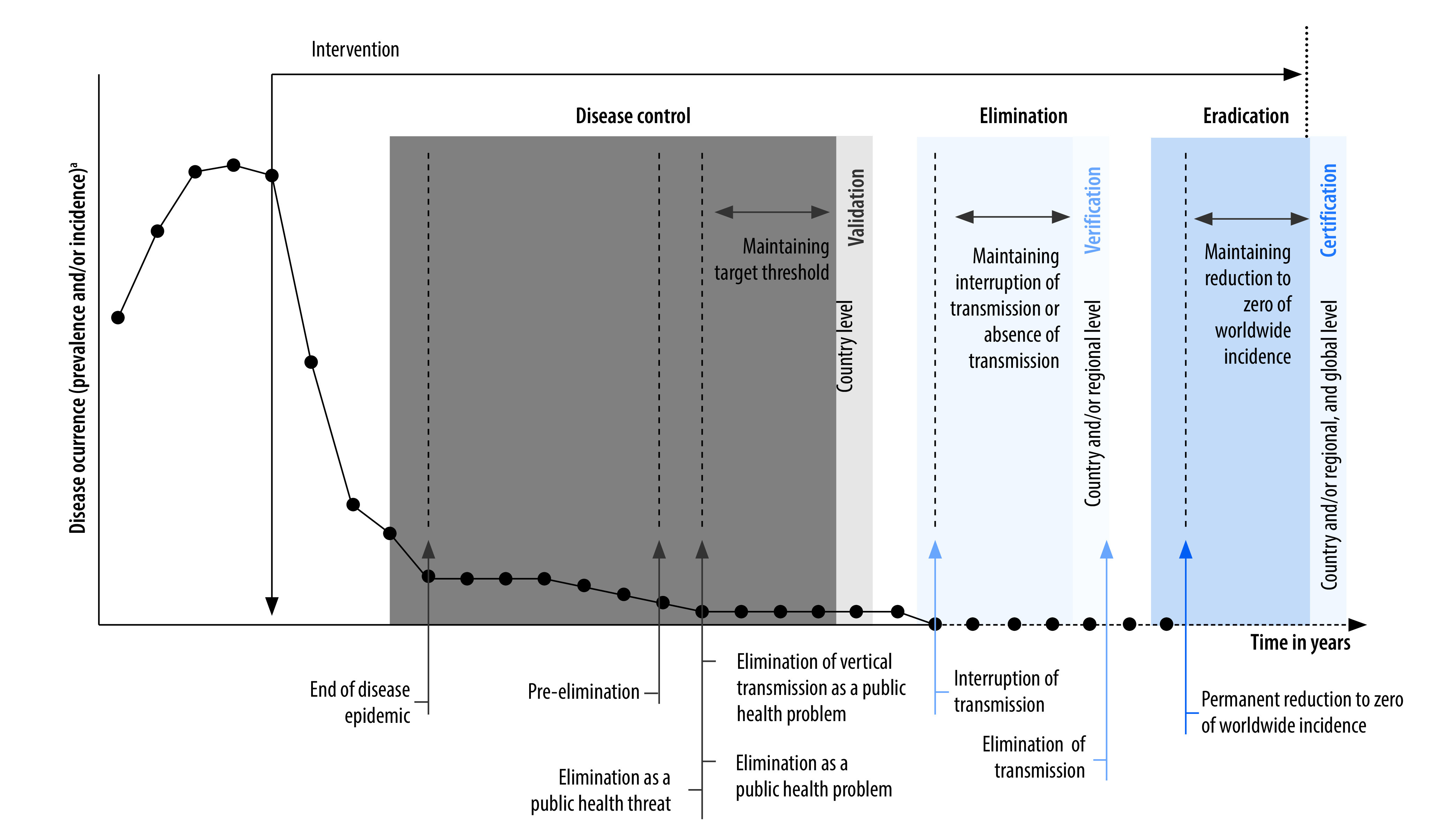
Graphical representation of disease control goals and their assessment processes

^a^ Prevalence (% and/or rate) and/or incidence (number of cases and/or rate), depending upon the type of goal and infectious condition.

Our review highlighted a chronological arrangement of tuberculosis goals, which provides a broad understanding of progression of goals where ending a disease epidemic and pre-elimination are a stepping-stone towards a higher goal of elimination as a public health problem. The goal of elimination as a public health threat was also identified as equivalent to the goal of elimination as a public health problem.^63^ In Fig. 3, we offer a graphical representation of the core concepts of disease control, elimination and eradication, with distinct goals along the spectrum of these concepts. We recommend a clear distinction be made between terms such as interruption of transmission, which is used once transmission is stopped, versus elimination of transmission, which is reserved for when the goal of interruption of transmission has been maintained years after achieving it. This distinction was clearly highlighted in one of the documents on onchocerciasis.^32^ Fig. 3 gives an overarching depiction of the disease control continuum and may not be applicable in its entirety to all infectious conditions. For some infectious conditions, which are not suitable for elimination or eradication, the end goal may just be an advanced level of control, for example, meningitis A or yellow fever. In addition, for some infectious conditions, interruption of transmission could be part of the overall strategy of elimination as a public health problem. For example, for Chagas disease, the aim is to interrupt transmission via four of six transmission routes to attain the overall goal of elimination as a public health problem.^22^

Importantly, the goal of elimination as a public health problem or threat needs to be reinforced, and probably rephrased, as an advanced level of control. This goal was created to secure the political impetus necessary for any concerted public health initiative.^74^ Achievement of this goal could create a false sense of success, and resource-constrained countries may divert their funds to other emerging problems, which could lead to continued undetected transmission resulting in undiagnosed cases and underreporting. One such example is leprosy, where evidence suggests that only 50% of cases are currently being detected in certain countries that have had otherwise met this goal.^75^ Further research is required to study the inadvertent consequences and costs of elimination and eradication, including the environmental impact of eliminating a vector.

We collected information on all interventions across the diseases, but to present our results concisely, we reduced interventions to five broad categories and included the respective interventions as footnotes in the online repository.^69^ For some conditions, such as, measles, rubella and congenital rubella syndrome, and meningitis A, we could not include all process targets, mostly related to laboratory testing and surveillance. Nonetheless, we included the essential process targets for these conditions. We excluded documents on diseases targeted only for disease control, such as Buruli ulcer and scabies, as the goal is in line with the definition of disease control defined at the Dahlem Workshop and does not require further clarification. Likewise, we excluded documents on coronavirus disease 2019 (COVID-19) as WHO has not targeted this disease for elimination. However, noteworthy is that WHO recently shifted its strategic objectives for COVID-19 from an emergency to a longer-term disease prevention and control response.^76^

We conducted our review in line with PRISMA guidelines and used a robust search strategy, covering a study period of more than 15 years. This method increases the validity of the results and allowed us to provide a comprehensive systematic review on disease control initiatives.

In conclusion, using standardized terminology and approaches across all disease control initiatives is imperative to realize disease control initiatives, particularly as countries focus on achieving the SDGs by 2030.
