# Corrigendum

**DOI:** 10.2471/BLT.23.101123

**Published:** 2023-11-01

In: Khawar L, Donovan B, Peeling RW, Guy RJ, McGregor S. Elimination and eradication goals for communicable diseases: a systematic review. Bull World Health Organ. 2023 Oct 1;101(10):649–665, 

the words *threshold* and *target* were used interchangeably. The word target should have been used throughout the article.

On page 657, Table 3 should read as follows:

**Table 3:** Disease endpoints and targets, by goal type and infectious condition

Goal type, by infectious condition	Disease endpoint	No. of targets	Type of target		
			No. or percentage	Rate	% reduction or fractional reduction
**Worldwide permanent reduction to zero**					
Dracunculiasis^16,19,22^	Cases	1	Zero	NR	NR
Polio^18,24^	Cases	1	Zero	NR	NR
Smallpox^14,15^	Cases	1	Zero	NR	NR
Yaws^16,17,20–23^	Cases	1	Zero	NR	NR
**Interruption of endemic transmission**					
Cholera^a,35^	Case	1	Zero, endemic, nationally	NR	NR
Leprosy^22,48,55,56^	New cases	4	Zero, new autochthonous cases, nationally. ≤ 62 500 annual new cases, globally	≤ 0.12/1 000 000 new cases with grade 2 disabilities, globally	90% reduction in new case rate in children, globally
Malaria^29,49,58,65^	Incidence	2	Zero indigenous cases, nationally	NR	90% reduction by 2030, globally
	Mortality	1	NR	NR	90% reduction by 2030, globally
Measles^28,37,38,45,50,51,59,60^	Cases	1	Zero, endemic, regionally	NR	NR
Rubella and congenital rubella syndrome ^28,37,38,45,50,51,59,60^	Cases	1	Zero, endemic, regionally	NR	NR
**Interruption of transmission**					
Human African trypanosomiasis (gambiense)^22,68^	Cases	1	Zero, nationally	NR	NR
Onchocerciasis^22,32^	Incidence	1	NR	Zero, nationally	NR
Rabies^40,41^	Cases in dogs	1	Zero canine cases, nationally	NR	NR
Schistosomiasis^22,67^	Incidence	1	NR	Zero	NR
**Elimination as a public health problem**					
Chagas disease^22^	Incidence	1	Zero,^b^ nationally	NR	NR
Human African trypanosomiasis (gambiense)^22,68^	Cases	2	< 2000 a year, globally	< 1/10 000 a year (in at-risk areas)	NR
Leprosy^22,48,55,56^	Prevalence	1	NR	< 1 case/10 000, nationally	NR
Lymphatic filariasis^22,25,36,57^	Prevalence	3	< 2% antigenaemia in all endemic areas^c^	NR	NR
			< 1% antigenaemia in all endemic areas^d^	NR	NR
			< 2% antibody prevalence in all endemic areas, nationally^e^	NR	NR
Maternal and neonatal tetanus^44,66^	Incidence	1	NR	< 1/1000 live births a year per district	NR
Rabies^22,40,41^	Mortality	1	Zero human deaths, nationally	NR	NR
Human African trypanosomiasis (rhodesiense)^22,68^	Cases	1	NR	< 1/10 000 a year per district	NR
Schistosomiasis^22,67^	Prevalence	1	< 1% of heavy-intensity infections, nationally^f^	NR	NR
Soil-transmitted helminths^22,46^	Prevalence	1	< 2% of moderate-to-heavy intensity infections in pre-school and school-aged children, nationally^g^	NR	NR
Trachoma^22,33,53^	Prevalence	2	< 0.2% TT in ≥ 15-year-olds, nationally	NR	NR
			< 5% TF in children, nationally	NR	NR
Tuberculosis (low-incidence countries)^h,27,31,52^	Incidence	1	NR	< 1 case/1 000 000, nationally	NR
Viral STIs – human papillomavirus-related cervical cancer^47,54^	Incidence	2	NR	4 cases/100 000 women-years, nationally	South-East Asian Region: reduce by one third by 2030
	Mortality	1	NR	NR	South-East Asian Region: reduce by one third by 2030
Visceral leishmaniasis^22,34^	Cases	1	NR	South-East Asian Region: < 1 case/10 000	NR
	Case fatality	1	For all countries other than in South-East Asian Region: < 1%	NR	NR
**Elimination of vertical transmission as a public health problem**					
Chagas^22,39^	Transmission rate	1	Zero, nationally	NR	NR
	Prevalence	1	≥ 90% children cured, nationally	NR	NR
Hepatitis B virus^39,43,62,64^	Prevalence	1	≤ 0.1% HBsAG prevalence in children < 5 years, nationally	NR	NR
	Transmission rate	1	< 2%, nationally^i^	NR	NR
HIV^39,43,62,64^	New cases	1	NR	≤ 50/100 000 live births, nationally	NR
	Transmission rate	1	< 5% and < 2% in breastfeeding and non-breastfeeding countries, respectively^j^	NR	NR
Syphilis^39,43,62,64^	New cases	1	NR	≤ 50/100 000 live births, nationally	NR
**Elimination as a public health threat**					
Viral hepatitis B (national level targets)^63,64^	Prevalence	2	≤ 0.5% HBsAg prevalence in children 0–5 years by 2025^k^	NR	NR
			≤ 0.1% HBsAg prevalence in children 0–5 years by 2030^k^	NR	Or 95% reduction by 2030^l^
	Incidence	2	NR	≤ 11/100 000 cases a year by 2025	NR
				≤ 2/100 000 cases a year by 2030	NR
	Mortality	2	NR	≤ 7/100 000 deaths a year by 2025	NR
				≤ 4/100 000 deaths a year by 2030	Or 65% reduction by 2030^l^
Viral hepatitis C (national level targets)^63,64^	Incidence	4	NR	≤ 13/100 000 cases a year by 2025	NR
				≤ 5/100 000 cases a year by 2030	Or 80% reduction by 2030^l^
				People who inject drugs: ≤ 3/100 a year by 2025	NR
				People who inject drugs: ≤ 2/100 a year by 2030	NR
	Mortality	2	NR	≤ 3/100 000 deaths a year by 2025	NR
				≤ 2/100 000 deaths a year by 2030	Or 65% reduction by 2030^l^
**Pre-elimination**					
Tuberculosis (low-incidence countries)^27,52^	Incidence	1	NR	< 10/1 000 000 cases by 2035, nationally	Or 90% reduction by 2035^l^
**End of disease epidemic**					
Cholera^35^	Mortality	1	≤ 9500 deaths by 2030	NR	Or 90% reduction by 2030, globally
	Outbreaks	1	Zero, uncontrolled	NR	NR
Meningitis A^61^	New cases	1	NR	NR	50% reduction by 2030, globally
	Mortality	1	NR	NR	70% reduction by 2030, globally
HIV^64^	New cases	6	All ages: ≤ 370 000/year by 2025, globally	0.05/1000 uninfected population a year	Or 75% reduction in the no. of new cases globally
			All ages: ≤ 335 000/year by 2030, globally	0.025/1000 uninfected population a year	Or 78% reduction in the no. of new cases globally^m^
			≤ 0–14 years: 20 000/year by 2025, globally	NR	Or 86% reduction, globally
			≤ 0–14 years: ≤ 15 000/year by 2030, globally	NR	Or 90% reduction, globally^m^
	Mortality	2	≤ 250 000 deaths/year by 2025, globally	NR	Or 63% reduction, globally
			< 240 000 deaths/year by 2030, globally	NR	Or >65% reduction, globally
	Mortality from comorbidity	2	≤ 110 000 deaths/year by 2025, globally^n^	NR	Or 48% reduction, globally
			≤ 55 000 deaths/year by 2030, globally^n^	NR	Or 74% reduction, globally
STIs (bacterial)^64^					
Syphilis	New cases	2	≤ 5 700 000/year by 2025, globally	NR	Or 20% reduction, globally
			≤ 710 000/year by 2030, globally	NR	Or 90% reduction, globally^m^
Gonorrhoea	New cases	2	≤ 65 800 000/year by 2025, globally	NR	Or 20% reduction, globally
			≤ 8 230 000/year by 2030, globally	NR	Or 90% reduction, globally^m^
STIs (overall): chlamydia, gonorrhoea, syphilis, trichomoniasis^64^	New cases	2	< 300 000 000/year by 2025, globally	NR	Or 20% reduction, globally
			< 150 000 000/year by 2030, globally	NR	Or 60% reduction, globally
Tuberculosis (high-incidence countries)^o,30,31^	Incidence	2	NR	< 20/100 000 cases by 2030, nationally	Or 80% reduction, globally^l^
			NR	< 10/100 000 cases by 2035, nationally	Or 90% reduction, globally^l^
	Mortality	2	NR	NR	90% reduction by 2030, globally
					95% reduction by 2035, globally
Yellow fever^42^	Outbreaks	1	Zero, uncontrolled	NR	NR
**Disease control**					
Chagas disease (oral route)^16^	NR	NA	NR	NR	NR
Cutaneous leishmaniasis^22^	No impact targets for disease endpoint	NA	NR	NR	NR
Schistosomiasis^26^	Prevalence	1	< 5% heavy-intensity infections^f^	NR	NR

AIDS: acquired immunodeficiency syndrome; HBsAG: hepatitis B surface antigen; HIV: human immunodeficiency virus; NA: not applicable; NR: not reported; STIs: sexually transmitted infections; TF: trachomatous inflammation – follicular; TT: trachomatous trichiasis.

^a^ The target is for 20 endemic countries to eliminate cholera.

^b^ Based on four of six transmission routes, that is, vectoral, transfusion, transplantation and congenital. The other two transmission routes are oral and laboratory accidents.

^c^ In areas where *Wuchereria bancrofti* is endemic and *Anopheles* or *Culex* is the main vector.

^d^ In areas where *Aedes* is the main vector.

^e^ In areas where *Brugia* spp. is endemic.

^f^ Heavy intensity of infections: *Schistosoma mansoni* (400 eggs/g faeces, *S*. *haematobium* (50 eggs/10 mL urine).

^g^ Caused by *Ascaris lumbricoides*, *Trichuris trichiura*, *Necator americanus* and *Ancylostoma duodenale.*

^h^ For details on the impact targets for the European region, see online repository.^69^

^i^ Additional target for countries using targeted timely hepatitis B vaccine birth dose.

^j^ Breastfeeding countries: countries where the benefits of breastfeeding in terms of child survival outweigh the risk of HIV transmission via breastfeeding. Non-breastfeeding countries: countries where women living with HIV who give birth are strongly recommended to avoid breastfeeding due to evidence of a risk of HIV transmission via breastfeeding.

^k^ Childhood prevalence is a proxy for incidence of chronic hepatitis B virus infection.

^l^ For viral hepatitis, the relative reduction targets are from a 2015 baseline; while the absolute targets take the baseline of 2020, however, the relative reduction targets from a 2020 baseline can be calculated from document ref# 64; likewise, for TB, the relative reduction targets are from a 2015 baseline.

^m^ From a 2020 baseline.

^n^ Mortality associated with causes related to tuberculosis, hepatitis B and C.

^o^ The targets can be adapted nationally depending on the baseline point.

Note: For shared targets with HIV, viral hepatitis and STIs, see online repository.^69^

